# The Impact of 90 Parkinson’s Disease-Risk Single Nucleotide Polymorphisms on Urinary Bis(monoacylglycerol)phosphate Levels in the Prodromal and PD Cohorts

**DOI:** 10.3390/ijms25042286

**Published:** 2024-02-14

**Authors:** Shuai Fang, Priscilla Ann Hweek Lee, Zejian Wang, Bo Zhao

**Affiliations:** Engineering Research Center of Cell and Therapeutic Antibody, Ministry of Education, School of Pharmacy, Shanghai Jiao Tong University, Shanghai 200240, China; shuaifang@sjtu.edu.cn (S.F.); hibarihweek@sjtu.edu.cn (P.A.H.L.)

**Keywords:** biomarker, GBA1, LRRK2, lysosome, PD, prodromal, urinary BMPs, 90 PD-risk SNPs

## Abstract

Parkinson’s disease (PD) is a common neurodegenerative disorder with a prolonged prodromal phase. Higher urinary bis(monoacylglycerol)phosphate (BMP) levels associate with LRRK2 (leucine-rich repeat kinase 2) and GBA1 (glucocerebrosidase) mutations, and are considered as potential noninvasive biomarkers for predicting those mutations and PD progression. However, their reliability has been questioned, with inadequately investigated genetics, cohorts, and population. In this study, multiple statistical hypothesis tests were employed on urinary BMP levels and sequences of 90 PD-risk single nucleotide polymorphisms (SNPs) from Parkinson’s Progression Markers Institution (PPMI) participants. Those SNPs were categorized into four groups based on their impact on BMP levels in various cohorts. Variants rs34637584 ^G/A^ and rs34637584 ^A/A^ (LRRK2 G2019S) were identified as the most relevant on increasing urinary BMP levels in the PD cohort. Meanwhile, rs76763715 ^T/T^ (GBA1) was the primary factor elevating BMP levels in the prodromal cohort compared to its T/C and C/C variants (N370S) and the PD cohort. Proteomics analysis indicated the changed transport pathways may be the reasons for elevated BMP levels in prodromal patients. Our findings demonstrated that higher urinary BMP levels alone were not reliable biomarkers for PD progression or gene mutations but might serve as supplementary indicators for early diagnosis and treatment.

## 1. Introduction

Parkinson’s disease (PD) is the most common movement disorder with progressive neurodegeneration [1,2,3]. It affects approximately 1% of the global population over the age of 60 and increases to 3–4% in those aged over 80 [4,5]. Aging, sex, genetics, and certain environmental influences heighten the risk of PD occurrence [6,7,8,9]. However, comprehensive understanding, essential for effective and curative treatments of the disease, remains elusive and diagnosis faces considerable challenges [10,11]. In recent decades, researchers have focused on the pathological processes of PD manifesting 10–20 years before clinical diagnosis [12,13]. The stage in which patients show various non-motor and/or subtle motor symptoms but are insufficient for PD diagnosis by current criteria is called prodromal PD, whose criteria were updated by the Movement Disorder Society in 2019 [13,14,15].

Numerous potential biomarkers for both prodromal and clinical PD have been identified, such as aggregated α-synuclein species in cerebrospinal fluid [16,17,18,19,20,21]. However, translating these findings into clinical practice remains challenging, primarily due to their partial PD specificity, complex and costly detection methods, and questionable accuracy [11,21,22,23,24]. Genetics plays a pivotal role in disease development, attracting significant attention [25]. This is not only due to the high mutation rates, particularly in familial PD cases, but also because of their crucial functions in cellular homeostasis, such as mitochondrial quality control and vesicle trafficking [26,27]. The most recent genome-wide association studies (GWAS) in PD have identified 90 significant risk loci from 7.8 million single nucleotide polymorphisms (SNPs) across 17 datasets of European ancestry samples [28]. However, it is worth noting that only 5–10% of patients suffer from monogenetic PD with Mendelian inheritance, while the majority of PD cases are sporadic, and the etiology remains enigmatic [29].

Bis(monoacylglycerol)phosphates (BMPs) are phospholipids found abundantly in the inner membranes of late endosomes and acidic vesicular transport complexes. They carry negative charges and provide docking and functional sites for lysosomal enzymes [30,31]. Elevated urinary BMP levels have been associated with (drug-induced) phospholipidosis and lysosomal storage disorders due to their strong correlation [31,32,33,34]. Urinary di-22:6 BMP was observed to reduce the presence of LRRK2 (leucine-rich repeat kinase 2) kinase inhibitors in rats and nonhuman primates [35]. Alcalay et al. reported higher urinary BMP (total di-18:1-BMP, total di-22:6-BMP, and their 2,2’ isoforms) levels in LRRK2 G2019S carriers, independent of PD status and sex [36]. Recently, both LRRK2 and GBA1 (glucocerebrosidase) mutations were found to be associated with higher urinary BMP concentrations in PD cohorts [37]. These findings collectively suggest the potential of urinary BMP levels as biomarkers for convenient screening of LRRK2 and GBA1 mutation carriers and mutation-targeted therapies for PD. Collecting urinary BMPs is noninvasive and better tolerated by patients compared to other biomarkers. However, no prior studies have conducted a comprehensive assessment to determine whether urinary BMP levels could serve as reliable biomarkers for PD states or the aforementioned gene mutations.

In this study, we aimed to fill this gap by investigating the impact of both the mutated and non-mutated variants of all 90 PD-risk SNPs on urinary BMP levels in both prodromal and PD cohorts. Datasets of urinary BMP levels (total di-18:1-BMP, total di-22:6-BMP, and 2,2’ di-22:6-BMP), variants of 90 PD-risk SNPs, and related age information from participants in three cohorts (healthy controls, prodromal, and PD cohorts) sourced from the Parkinson’s Progression Markers Initiative (PPMI, https://www.ppmi-info.org/) (accessed on 12 August 2022) were collected [38].

We conducted a series of statistical analyses, including Levene’s test, one-way Analysis of Variance (ANOVA), Student’s *t*-test, Mann–Whitney U test, random forest regression, and partial correlation analysis, to evaluate the impact of variants of 90 PD-risk SNPs on urinary BMP levels. Our findings unveiled a multifaceted relationship between gene mutations and urinary BMP levels, furnishing valuable insights for both disease research and clinical applications.

## 2. Results

### 2.1. The Prodromal Cohort Had Higher Urinary BMP Levels than the PD and Healthy Controls

**Figure 1:**
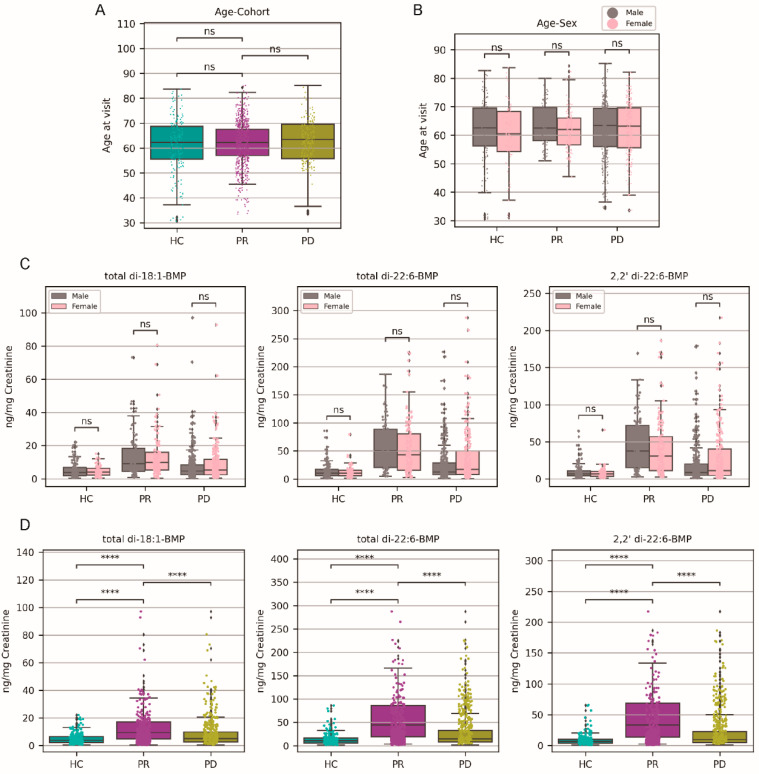
The three cohorts of Parkinson’s disease with similar characteristics had different urinary BMP levels. (**A**,**B**) Age homogeneity analysis among cohorts and sex showed no age differences. The age numbers of participants were grouped by cohorts (**A**) or sex (**B**) after Box–Cox transformation and then Levene’s test and the F-test within one-way ANOVA were employed to validate the assumption of homogeneity of variances. (**C**) Sex-related urinary BMP analysis within each cohort showed no sex differences. The data of urinary BMP levels in each cohort underwent Box–Cox transformation initially and were then examined using appropriate statistical tests such as *t*-tests and Mann–Whitney U (M–W) tests, contingent on the adherence of transformed data to normality assumptions. (**D**) Cohort-based urinary BMP analysis showed higher urinary BMP levels were found in the prodromal and PD cohort than in the healthy control, and the BMP levels were higher in the prodromal than in the PD cohort. The box plots and strip plots showed the aggregate and individual data of the three urinary BMP levels within each cohort. Significant statistical markers were annotated according to *p*-values calculated by the Mann–Whitney U ‘two-sided’ test after Box–Cox transformation of BMP levels. The threshold for significance in pairwise comparisons adhered to Bonferroni correction was α = 0.016. Note: ns—non-significant; ****—*p* < 0.0001.

Project 145 of the PPMI (Parkinson’s Progression Markers Initiative) examined the urinary levels of three BMPs (biomarker phospholipids) in a group of 1248 participants, including total di-18:1-BMP, total di-22:6-BMP, and 2,2’ di-22:6-BMP. Simultaneously, Project 118 sequenced 90 SNPs (Single Nucleotide Polymorphisms) associated with Parkinson’s disease in a cohort of 1070 participants. We amalgamated these two datasets, revealing that 954 participants underwent both urinary BMP and DNA testing. Our study encompassed three distinct cohorts: healthy controls (HC), individuals in the prodromal stage (PR), and those diagnosed with Parkinson’s disease (PD). More detailed information of age and sex characteristics, and the overall urinary BMP features of participants are listed in Table 1. Please refer to Table S1 for detailed data of participants on sex, age, cohort, BMP levels, and sequencing results of 90 PD-risk SNPs. Importantly, participants of different cohorts and sex exhibited a similar age range (Figure 1A,B), and the urinary BMP levels were similar between sex in each cohort (Figure 1C).

To examine the differences in BMP levels among the three cohorts with varying Parkinson’s disease states, we employed hypothesis tests to assess significance. Since the BMP-level data did not adhere to a normal distribution, both before (*p* < 0.0001) and after (*p* = 0.0153) Box–Cox transformation—a common method for normalizing non-normally distributed variables—we applied the non-parametric Mann–Whitney U test (abbreviated as M–W test) to analyze the BMP level data. The Mann–Whitney test (M–W) brought forth compelling evidence by demonstrating significantly elevated levels of all three urinary BMPs within both the prodromal (PR) and Parkinson’s disease (PD) cohorts, in comparison to the healthy controls (HC) (Figure 1D). This observation was in line with earlier findings. However, an intriguing and unexpected result was the PR cohort exhibiting even higher urinary BMP levels than the PD cohort (Figure 1D). In-depth investigation and analysis of this unanticipated outcome would be needed.

### 2.2. The Impact of Variants of 90 PD-Risk SNPs Varied in Different Cohorts of PD

Given the importance of genetics in PD, and that 90 PD-risk SNPs were verified by Nalls et al. [28] in the latest GWAS, we focused on comprehending how the variants of the 90 PD-risk SNPs impacted urinary BMP levels. To achieve this, we tried to discover the genetic factors relevant to elevated urinary BMP levels within prodromal and PD cohorts, with particular emphasis on the prodromal cohort. We noticed that the increasing trend of the BMP levels in the order of HC < PD < PR was observed across all participant groups sharing the same SNP variant (data not shown). This pattern constrained the BMP levels comparison among the three cohorts based on SNP variants and suggesting that the factors contributing to BMP level variations were intrinsic to each respective cohort. To assess the influence of SNP variants on different PD cohorts, we conducted separate investigations for each of the 90 PD-risk SNPs. Specifically, we grouped participants from each cohort—healthy control, prodromal, and PD—based on the variant they carried. Subsequently, we compared urinary BMP levels between any two variants of each SNP. Among these 90 SNPs, 85 of them exhibited three variants: the variant without mutations (V0), the variant with a mutation on one of the alleles (V1), and the variant with mutations on both alleles (V2). This resulted in three sets of comparisons: V0:V1, V0:V2, and V1:V2. The remaining 5 SNPs had only two variants, V0 and V1, leading to a single comparison: V0:V1.

**Figure 2:**
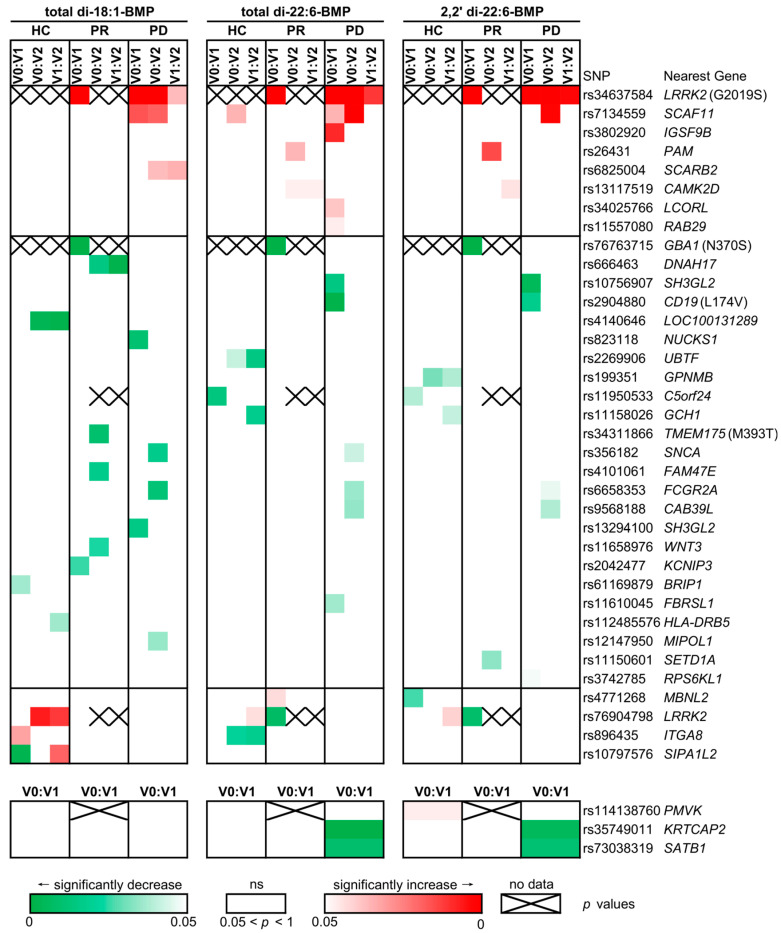
The heatmap illustrates the significance of *p*-values across various variants of each SNP within each cohort. The *p*-values were firstly computed using a M–W test under the ‘less’ hypotheses, and those with *p* ≤ 0.05 indicated significant higher BMP levels in the latter variant carriers than in the former variant carriers. Then, the M–W test under the ‘greater’ hypotheses was taken oppositely to compute the *p*-values for decreasing BMP level in the former compared to the latter. All *p*-values were subjected to Bonferroni correction for adjustment. The heatmap adopts adjusted *p*-values instead of an adjusted significance level (α = 0.0167 for SNPs with 3 variants and α = 0.05 for SNPs with 2 variants) to better visualize the significance of SNPs with varying numbers of variants in a single figure. The heatmap blocks representing the significant *p*-values corresponding to the ‘less’ and ‘greater’ hypotheses were colored in gradient red (indicating a significant increase) and green (significant decrease), respectively. The colored blocks under different hypotheses were then integrated into a unified heatmap for intuitive representation. The nearest gene list referred to the publication by Nall et al., with a protein mutation site displayed following the gene name when the SNP mutation would result in a missense variant. Note: HC—healthy controls; PR—the prodromal cohort; PD—the PD cohort; V0—the non-mutated homozygous variant; V1—the mutated heterozygous variant; V2—the mutated homozygous variant; ns—non-significant; no data—comparison was not feasible due to one or both groups having fewer than 3 samples.

We calculated *p*-values using the Mann–Whitney U test, employing one-sided (greater or less, successively) checks to verify either a decrease or an increase in urinary BMP levels. Based on the observed changes in BMP levels related to SNP mutations, the 90 PD-risk SNPs were categorized into four distinct classes: those whose mutations related to (1) increase or (2) decreased levels of the three BMPs, (3) had varying impacts across different cohorts and with different mutations, and (4) had no significant effect on urinary BMP levels in any cohort (Figure 2, data not shown for the fourth class). The first class comprised nine SNPs, such as rs34637584 (LRRK2), rs7134559 (SCAF11), rs3802920 (IGSF9B), rs26431 (PAM), and rs6825004 (SCARB2) (Figure S1A–D for the latter four SNPs). The second class consisted of 26 SNPs, such as rs76763715 (GBA1), rs666463 (DNAH17), rs10756907 (SH3GL2), rs2904880 (CD19), rs35749011 (KRTCAP2), and rs73038319 (SATB1) (Figure S1A–E for the latter 5 SNPs). The third class displayed fluctuating trends in BMP levels; however, if only prodromal and PD cohorts were considered, the rs4771268 (MBNL2) and rs76904798 (LRRK2) were allocated to the first and second class, respectively, and rs896435 (ITGA8) and rs10797576 (SIPA1L2) were considered to be in the fourth class (Figure S3A–D). Notably, it is worth mentioning that nucleotide mutations in the majority of these SNPs did not correspond to protein mutations (substitution sites were indicated in brackets on the right of Figure 2). For detailed *p*-value pertaining to each SNP, please refer to Table S2.

### 2.3. Variants rs34637584 ^G/A^ and rs34637584 ^A/A^ (LRRK2 G2019S) Were the Primary Genetic Factors for Elevated Urinary BMP Levels in the PD Cohort than in Healthy Controls

**Figure 3:**
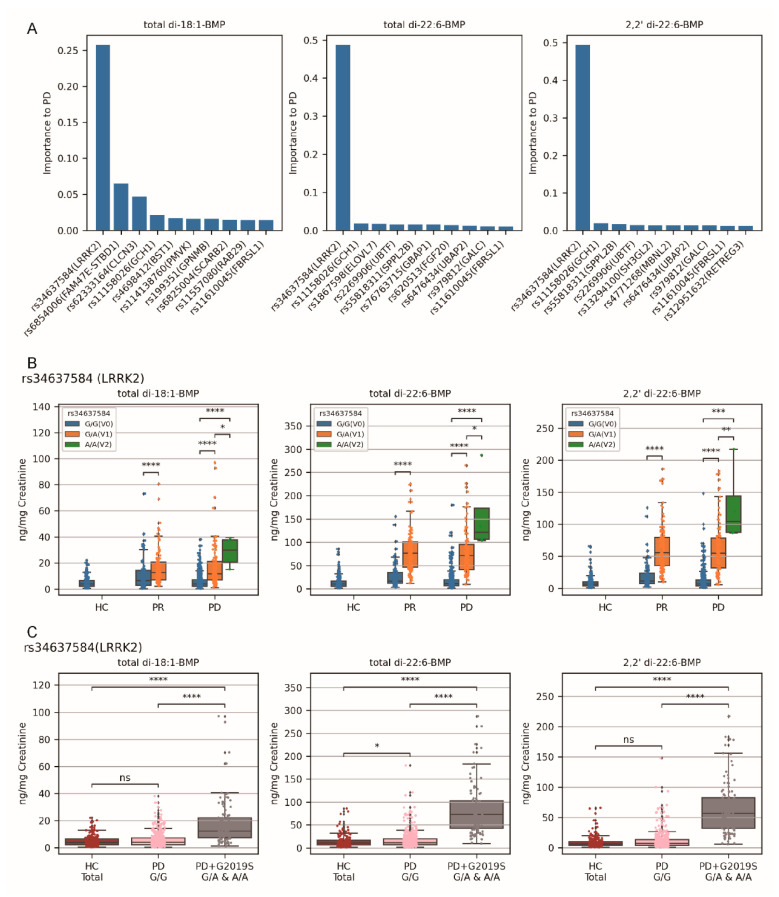
Influence of rs34637584 (LRRK2) variants on urinary BMP levels in the PR and PD cohorts. (**A**) Random forest regression analysis identified rs34637584 (LRRK2) as the primary SNP whose variants account for urinary BMP levels in the PD cohort. To calculate the weighted importance values of 90 PD-risk SNPs, the mutation numbers of 90 SNPs were used as independent variables and the value of one of the three BMP levels was used as one continuous dependent variable. The inherent algorithm of random forest regression calculated their importance scores, and then the top 10 SNPs were identified. (**B**) The rs34637584 (LRRK2) variants increased the three urinary BMP levels in each cohort in the order of G/G < G/A < A/A. Participants of each cohort were grouped by the variant they were carrying, and their BMP levels after Box–Cox transformation were subjected to comparison using the M–W test with the ‘less’ hypotheses when the number of participants in both groups were at least 3. (**C**) The variants rs34637584 ^G/A^ and rs34637584 ^A/A^ (LRRK2 G2019S) increased urinary BMP levels in the PD cohort, and the levels in the PD cohort with rs34637584 ^G/G^ were not affected compared to HC (the urinary BMP levels of HC total were treated as standard baselines). Participants were grouped as indicated, pairwise comparisons of BMP levels were conducted using M–W test with ‘two-sided’ hypotheses. All the computed *p*-values were adjusted with Bonferroni correction. Note: HC—healthy controls; PR—the prodromal cohort; PD—the PD cohort; V0—the non-mutated homozygous variant; V1—the mutated heterozygous variant; V2—the mutated homozygous variant; ns—*p* > 0.05; *—*p* < 0.05; **—0.05 < *p* < 0.001; ***—0.0001 < *p* < 0.001; ****—*p* < 0.0001.

The higher urinary BMP levels in the PD cohort than in the HC were observed in Figure 1D, and the variants of many SNPs associated with increased BMP levels in the PD cohort in Figure 2. To address the primary SNP variant, random forest regression analysis was conducted to identify the top 10 SNPs (Figure 3A). Among them, rs34637584, whose nucleotide mutation G to A corresponds to missense protein mutation LRRK2 G2019S, exhibited the highest importance (see Figure S4A and S4B for rs6854006 and rs62333164, respectively). It was therefore considered as the primary SNP whose variants influencing the levels of all the three BMPs within the PD cohort.

For rs34637584 (LRRK2), three variants were identified in participants of the PD cohort, G/G (non-mutated homozygous, V0), G/A (compound heterozygous, V1), and A/A (mutated homozygous, V2). The baseline of all three BMP levels increased in the order of G/G < G/A < A/A in the PD cohort (Figure 3B). To assess the impact of the G2019S mutation on the PD cohort, participants carrying G/A and A/A variants were separated. The results showed that PD patients with rs34637584 ^G/G^ displayed no significant differences in all three BMP levels compared to healthy controls, while the differences between the PD + G2019S group (comprising G/A and A/A) and healthy controls or PD-G/G remained substantial (*p* < 0.0001) (Figure 3C). These findings confirmed that rs34637584 ^G/A^ and rs34637584 ^A/A^ (LRRK2 G2019S) were the primary genetic factors contributing to higher urinary BMP levels in the PD cohort compared to healthy controls. Furthermore, the prodromal cohort was also affected by LRRK2 G2019S, consistent with the findings of Merchant et al.’s work [37] when we presented the data in a similar format to theirs (Figure S4C).

### 2.4. Variant rs76763715 ^T/T^ (GBA1) Emerged as the Primary Factors to Elevated Urinary BMP Levels in the PR Cohort Compared to PD Cohort

**Figure 4:**
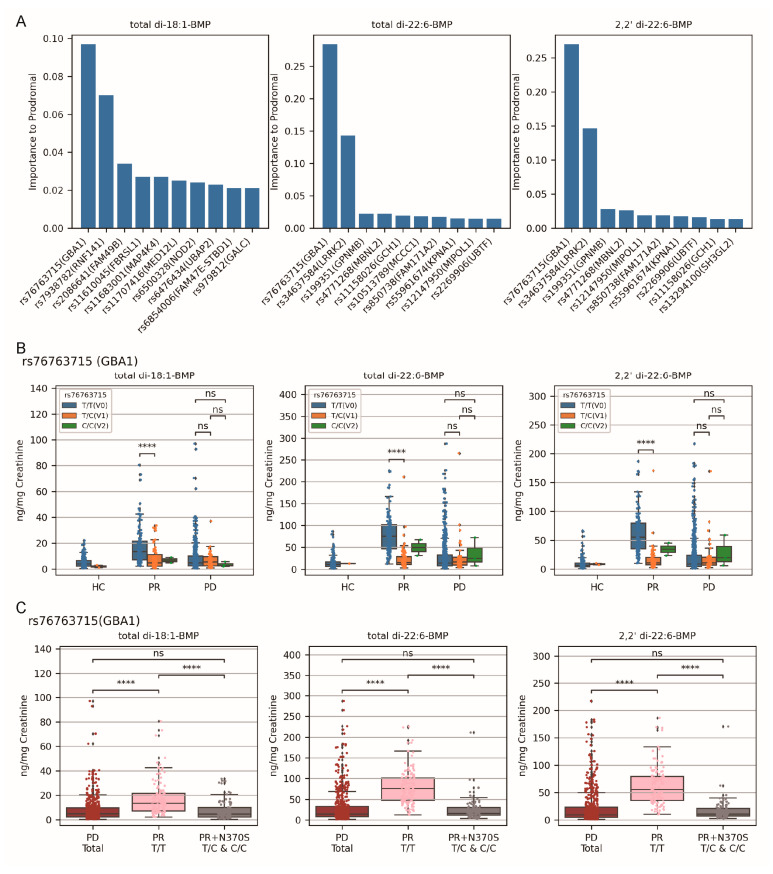
Influence of rs76763715 (GBA1) variants on urinary BMP levels in PR and PD cohorts. (**A**) Random forest regression analysis identified rs76763715 (GBA1) as the primary SNP whose variants account for urinary BMP levels in the PR cohort. With the mutation numbers of 90 PD-risk SNPs as independent variables and the level of one of the three BMPs as a continuous dependent variable in the PR cohort, the inherent algorithm of random forest regression calculated the importance scores of those SNPs, and then the top 10 were identified. (**B**) The V0 variant rs76763715 ^T/T^ (GBA1) unexpectedly increased all three urinary BMP levels in the PR cohort, and the PD cohort was not affected by those variants. Participants of each cohort were grouped by the rs76763715 variant they were carrying, and their BMP levels after Box–Cox transformation were compared by M–W test with the ‘greater’ hypotheses when the numbers of participants in both groups were at least 3. (**C**) The variant rs76763715 ^T/T^ (GBA1) increased the urinary BMP levels in the PR cohort, and the levels of the PR cohort with variants rs76763715 ^T/C^ and rs76763715 ^C/C^ (GBA1 N370S) were comparable to those in the PD cohort (the urinary BMP levels of PD total were treated as standard baselines). Participants were grouped as indicated and their BMP levels were compared pairwise by M–W test with ‘two-sided’ hypotheses. All computed *p*-values were adjusted with Bonferroni correction. Note: HC—healthy controls; PR—the prodromal cohort; PD—the PD cohort; V0—the non-mutated homozygous variant; V1—the mutated heterozygous variant; V2—the mutated homozygous variant; ns—*p* > 0.05; ****—*p* < 0.0001.

Similarly, random forest regression analysis was employed to identify the primary SNPs for higher urinary BMP levels in the prodromal cohort. Among the 90SNPs, rs76763715 was found to be most relevant in elevating all three BMPs within the PR cohort (Figure 4A). This SNP also exhibited three variants: T/T (V0), T/C (V1), and C/C (V2), and the nucleotide mutation T to C corresponds to missense protein mutation GBA1 N370S (also known as N409S). It was intriguing that participants carrying rs76763715 ^T/T^ demonstrated the highest BMP levels compared to rs76763715 ^T/C^ and rs76763715 ^C/C^ in the prodromal cohort, indicating the mutation of rs76763715 (GBA1) decreased the BMP levels in this cohort (Figure 4B). However, the PD cohort remained unaffected by this mutation (Figure 4B). The second and third SNPs pertaining to total di-18:1-BMP level, rs7938782 (RNF141) and rs2086641 (FAM49B), exhibited no significance to any BMPs under M–W test (Figure S5A,B). This discrepancy might be attributed to distinct calculation methods and research objectives. The second SNP associated with total di-22:6-BMP and its 2, 2′-isoform was rs34637584 (LRRK2), and its variant, rs34637584 ^G/A^, increased the levels of all three BMPs in the prodromal cohort, as shown in Figure 3B.

Considering the results in Figure 4B in conjunction with Figure 1D, it was plausible to suggest that rs76763715 ^T/T^ (GBA1) played a pivotal role in driving higher urinary BMP levels in the prodromal cohort compared to the PD cohort. This conclusion was further substantiated by the fact that after isolating the T/T carriers within the prodromal cohort, the remaining prodromal participants (T/C and C/C, GBA1 N370S) exhibited no significant difference in BMP levels compared to the PD cohort (Figure 4C). However, our results did not conflict with the findings of Merchant et al. [37]. When we organized our patients into the same groups as they did, we arrived at the same conclusion: PR and PD patients carrying the GBA1 N370S (rs76763715 ^T/C^ and rs76763715 ^C/C^) demonstrated higher BMP levels than the HC group (rs76763715 ^T/T^) (Figure S5C).

### 2.5. The Overall Significance of Factors on Urinary BMP Levels

By conducting a nonparametric hypothesis test, specifically the Mann–Whitney U test, we verified that age and sex had no significant impact on urinary BMP levels across the cohorts. Instead, the analysis highlighted the pivotal roles of rs34637584 (LRRK2) and rs76763715 (GBA1) among the 90 PD-risk SNPs. To corroborate these findings, we performed a partial correlation analysis involving age, sex, cohort (HC, PR, and PD), rs34637584 (LRRK2), rs76763715 (GBA1) and the urinary BMP levels by SPSS (Table 2). Once again, the results underscored the substantial influence of rs34637584 (LRRK2) and rs76763715 (GBA1) on the three urinary BMPs, while diminishing the significance of sex and age as contributors to these BMP levels.

### 2.6. Pathway and Process-Enrichment Analysis for Alterations in BMP Level

**Figure 5:**
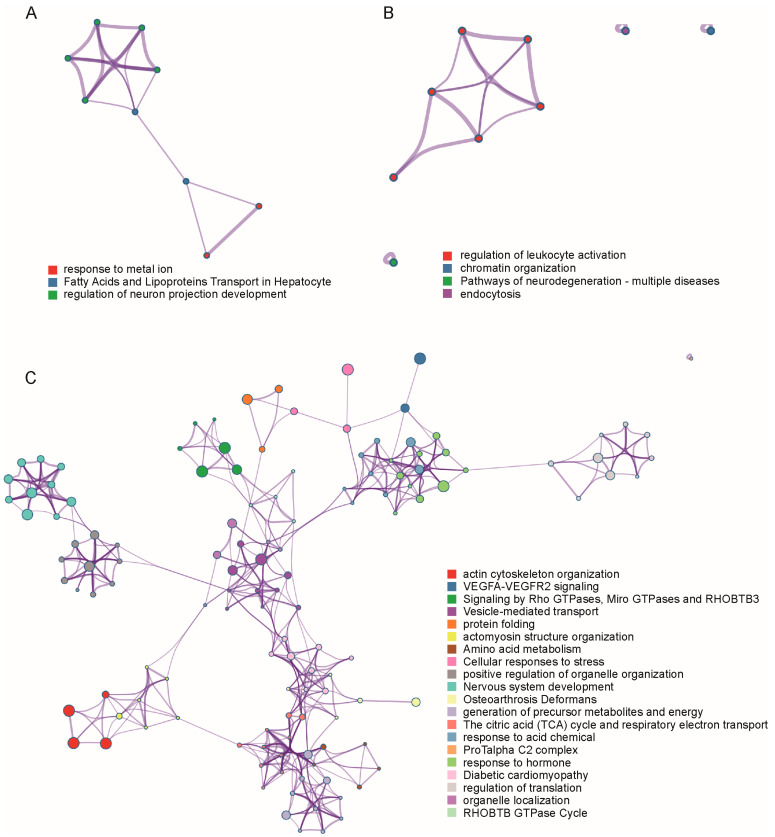
GO and KEGG pathway-enrichment network of (**A**) the nearest genes of SNPs with mutations that increased the urinary BMP levels, (**B**) the nearest genes of SNPs with mutations that decreased the urinary BMP levels, and (**C**) the gene encoding proteins with significantly more than 2-fold or less than 0.5-fold change in abundance ratio, as determined by TMT proteomics analysis between PR and PD patients carrying the rs76763715 ^T/T^ (GBA1) variant. The network was visualized using Cytoscape [40], where each node represented an enriched term. Nodes were colored by their cluster ID, with nodes of the same cluster ID typically located in close proximity. To depict the relationships between terms more effectively, a subset of enriched terms was selected and displayed as a network plot, where terms with a similarity of >0.3 were connected by edges. The terms with the best *p*-values from each of the 20 clusters were selected, with the constraint of limiting each cluster to no more than 15 terms and a total of 250 terms overall.

To explore the potential pathways associated with variations in urinary BMP levels due to SNP variants, a single gene list analysis employing Gene Ontology (GO) and Kyoto Encyclopedia of Genes and Genomes (KEGG) enrichment was conducted via Metascape [39]. Specifically, this analysis was applied to the nearest genes of the first and second classes of SNPs. The outcomes of pathway and process enrichment revealed that gene mutations linked to elevated urinary BMP levels were likely implicated in processes related to the response to metal ion, fatty acid, and lipoprotein transport in hepatocyte and regulation of neuron projection development (Figure 5A). Conversely, mutations leading to reduced BMP levels were predominantly associated with processes such as leukocyte activation, chromatin organization, pathways of neurodegeneration and multiple diseases, and endocytosis (Figure 5B).

In an effort to discern the underlying factors contributing to the increase in urinary BMP levels in individuals carrying the rs76763715 ^T/T^ (GBA1) variant during the prodromal stage, which subsequently decreased upon PD diagnosis, we conducted a comparative analysis of protein expression. Proteomic data were obtained from the PPMI project 150.2, in which protein lysates were collected from iPSC (induced pluripotent stem cell)-derived dopaminergic neurons at day 65 for Tandem Mass Tag (TMT) proteomics with three replicates (Table S3). The comparison involved a prodromal patient and a PD patient, both of whom carried rs76763715 ^T/T^ (GBA1). Proteins exhibiting a more than 2-fold or less than 0.5-fold change in abundance ratio and demonstrating statistical significance (*p* ≤ 0.05) according to a Student’s *t*-test between prodromal and PD patients were selected for pathway-enrichment analysis using Metascape (Figure 5C). The enriched pathways, such as actin cytoskeleton organization, vesicle-mediated transport, positive regulation of organelle organization, nervous system development, response to acid chemical, and organelle localization, pointed to significant changes in material-transport pathways between organelles in these two biospecimens, which might be the reason for higher urinary BMP levels in the prodromal than in the PD patients carrying the rs76763715 ^T/T^ (GBA1) variant.

## 3. Discussions

PD has long been the subject of extensive research, with a focus on its characterization as a neurodegenerative disease marked by dysfunctions in the ubiquitin–proteasome system and mitochondria [41,42]. However, emerging evidence points to PD’s significant association with lysosomal dysfunction and autophagy [43,44,45]. Among the 90 PD-risk SNPs, rs34637584 and rs76763715 stood out as particularly intriguing due to their profound influence on urinary BMP levels in PD patients compared to the others. Nucleotide mutations rs34637584 G to A and rs76763715 T to C correspond to protein missense variants LRRK2 G2019S and GBA1 N370S, respectively. LRRK2 (leucine-rich repeat kinase 2) mutations pose PD risk factors for both familial and sporadic cases [46]; it plays roles in synaptic vesicle endocytosis, trans-Golgi network maintenance and sorting, vesicular trafficking, and autophagy [47]. The LRRK2 G2019S mutation enhances its kinase activity, leading to lysosomal enlargement, potentially due to alterations in lysosomal iron channels [48]. LRRK2 G2019S disrupts the autophagy-lysosomal pathway in mouse neurons [49]. GBA1 mutations are also involved in sporadic PD [50]. GBA1 encodes the lysosomal enzyme glucocerebrosidase (GCase), responsible for hydrolyzing glucosylceramide (GlcCer) into glucose and ceramide (Cer) [51]. GBA1 mutations frequently give rise to lysosomal storage disorders like Gaucher disease (GD) [52]. GBA1 N370S mutations diminish GCase activity, leading to GlcCer accumulation and lysosomal enlargement [53]. Furthermore, GBA1 N370S mutations cause cholesterol buildup, impair autophagosome-lysosome function, and promote apoptosis [54].

The precise mechanisms governing the secretion of BMPs into urine remain unclear. As indicated by GO and KEGG pathway-enrichment results from proteomics, one hypothesis posits that, under the dysfunction of endolysosomes, cells generate endosome-derived extracellular vesicles containing BMPs to alleviate the accumulation of undigested materials and mitigate the heightened pressure within enlarged lysosomes [55]. As the disease advances, the buildup of materials within endolysosomes extends to other cellular membranes through organelle interactions [56]. In patients with rs76763715 ^T/T^ (GBA1), dysfunctions may manifest even during the prodromal stage, concurrent with other relevant genetic mutations. Similar to other chronic diseases, upregulated autophagy leading to lysosomal degradation in early PD stages acts as a protective mechanism against the aggregation of harmful proteins. Consequently, cells harboring rs76763715 ^T/T^ (GBA1) at the prodromal stage maintain the capacity to secrete BMPs for self-preservation, resulting in elevated BMP levels. However, the persistent accumulation of undigested substances and enlarged endolysosomes jointly contribute to autophagy inhibition and exosome secretion suppression, leading to cellular demise and irreparable damage. Continuous and intensive BMP secretion at an early stage gradually alters the composition of endosome-derived extracellular vesicles, with severe damage and substantial cell loss in later stages contributing to decreased BMP levels. Successive cycles of compensation and decompensation result in patients with rs76763715 ^T/T^ (GBA1) in the PD phase displaying lower BMP levels than those observed in the prodromal phase. Subsequently, the buildup of materials in lysosomes exacerbates the cellular condition over time, inducing additional dysfunctions, such as endoplasmic reticulum stress, mitochondrial dysfunction, and neuronal damage, ultimately culminating in PD development. Nevertheless, carefully designed studies would be needed to confirm this argument.

Additionally, rs76904798 and rs34311866, are the other two SNPs which are very common in PD and attract much attention. Rs76904798 is a non-coding polymorphism of LRRK2 [57]. Its variant, rs76904798 ^T/T^, significantly increases the total di-18:1-BMP level in HC, and the variant rs76904798 ^C/T^ decreases the total and 2, 2′ di-22:6-BMP levels in the PR, but they made no differences in the PD cohort (Figure 2). Based on the fact that its impact to BMP levels varied among cohorts, it was categorized into the third group of SNPs. Obviously, it was not the reason for higher BMP levels in PD than in HC, and its inconsistent impact on the three BMP levels indicated it not the main reason for higher levels of all BMPs in PR than in PD. Rs34311866 is a missense SNP in transmembrane protein 175 (TMEM175). TMEM175 is a lysosomal ion channel protein, and the missense variants of its two SNPs, rs34311866 (M393T) and rs34884217 (Q65P), are highly related to PD [58]. The former was identified to increase the risk of PD, while the latter was just the opposite [59]. It might be the reason that the latter SNP, rs34884217, was not identified in the GWAS of Nalls et al. [28], which aimed to find the PD-risk signals. Therefore, it was not in our list to be analyzed. The former SNP, rs34311866, was also included in this study. Its variant, rs34311866 ^C/C^, showed a decrease in the level of total di-18:1-BMP in the prodromal cohort (Figure 2). However, the variants of TMEM175 did not contribute as much as LRRK2 and GBA1.

## 4. Materials and Methods

### 4.1. Data Source

Datasets comprising three urinary BMP levels (total di-18:1-BMP, total di-22:6-BMP, and 2,2’ di-22:6-BMP) and sequences for 90 PD-risk SNPs were extracted from the file titled ‘Current_Biospecimen_Analysis_Results.csv’ obtained from the PPMI. These datasets originated from PPMI projects 145 and 118, respectively. Project 145 encompassed 1248 participants, consisting of 193 healthy controls (HC), 409 individuals in the prodromal stage (PR), and 646 patients diagnosed with Parkinson’s disease (PD), with a male/female ratio of 1.20. Project 118 included 1070 participants, comprising 195 HC, 282 PR, and 593 PD patients, with a male/female ratio of 1.36. Age data, extracted from the ‘Age_at_visit.csv’ file in PPMI, was employed to align urinary BMP levels with participants’ age at the time of their visit. The proteomics data were extracted from patient No.50184 (PR) and No.51971 (PD) in PPMI project 150.2, both patients carrying rs34637584 ^G/A^ and rs76763715 ^T/T^.

Here, as PPMI declared, HC were “participants with no neurologic disorder and no first-degree relative with PD”, PR included “participants who are at risk of Parkinson’s based on clinical features, genetic variants, or other biomarkers”, and PD referred to “Participants with early, untreated sporadic Parkinson’s disease or PD with a pathogenic genetic variant(s)”. Please visit https://www.ppmi-info.org/study-design/study-cohorts (accessed on 12 August 2022) for details.

### 4.2. Hypothesis Tests for Significance Check

The datasets were initially assessed for their distribution. Neither age nor urinary BMP levels followed a normal distribution (*p* < 0.0001). Consequently, Box–Cox transformation was applied to modify their distribution [60]. After transformation, the age dataset conformed to a normal distribution (*p* = 0.7829). It was subsequently appropriately grouped and assessed for equal variance using Levene’s test and then the F-test in one-way ANOVA [61]. Urinary BMP levels, not following a normal distribution after Box–Cox transformation (*p* = 0.0153), underwent non-parametric hypothesis tests for all significance checks. Data on urinary BMP levels were grouped as required, such as by cohorts or SNP variants, and compared using the Mann–Whitney U (M–W) test with one-sided (‘greater’ or ‘less’) or ‘two-sided’ hypotheses [62]. The *p*-values computed for pairwise comparisons were subjected to Bonferroni correction due to multiple comparisons [63]. It is worth noting that the urinary BMP levels of the HC cohort were treated as standard baselines when HC was compared with others.

### 4.3. Heatmap for p-Values of Impact of SNPs’ Variants on BMP Levels

For each SNP, participants of each cohort were grouped by the variant they were carrying. Subsequently, pairwise comparisons of their urinary BMP levels were conducted utilizing the Mann–Whitney U test, first under the ‘greater’ hypothesis (indicating a decrease in BMP levels) and then under the ‘less’ hypothesis (indicating an increase). The three variants of a SNP—non-mutated homozygous, compound heterozygous (with a mutation on one allele), and mutated homozygous (with mutations on both alleles)—were marked as V0, V1, and V2, respectively. The calculation of *p*-values occurred exclusively when the number of participants in both compared groups ≥ 3. These derived *p*-values underwent rigorous Bonferroni correction due to multiple comparisons. Adjusted *p*-values reflecting statistical significance in the context of ‘greater’ or ‘less’ hypotheses were colored in gradient green or red by Excel, respectively, and then integrated into a unified heatmap for intuitive representation.

### 4.4. Assessment of Factors Influencing Urinary BMP Levels

To elucidate the most significant genetic contributors to patients’ urinary BMP levels while minimizing the complexity of the model, we conducted a comprehensive analysis using random forest regression [64]. This approach was employed to quantify the importance (weight) of the 90 PD-risk SNPs in relation to BMP levels. The 90 SNPs were treated as independent variables, and each of the three BMP levels were regarded as a continuous dependent variable. To facilitate numerical computations, the SNP variants—V0, V1, and V2—were numerically encoded as 0, 1, and 2, corresponding to the number of mutations. Separate random forest regression analyses were performed for each BMP, producing weighted importance values for all 90 SNPs. These importance scores were used to rank the SNPs in descending order based on their influence on each urinary BMP. The ten most influential SNPs for each BMP within both prodromal and PD cohorts were selected for visualization in bar charts. Additionally, a partial correlation analysis was executed using the dataset, encompassing variables such as sex, age, cohort, rs34637584 (LRRK2), rs76763715 (GBA1), and the three urinary BMP levels (total di-18:1-BMP, total di-22:6-BMP, and 2,2’ di-22:6-BMP). Sex and age were considered as covariates in this analysis. The correlation and significance between any two variables were meticulously calculated using the Statistical Package for the Social Science (SPSS 22) software.

### 4.5. Proteomics Data Analysis

The proteins profiles of prodromal (PR) and PD patients, both carrying rs34647584 ^G/A^ and rs76763715 ^T/T^, from three distinct repeats were aligned to create a consolidated dataset containing common proteins. To identify proteins that exhibited significant changes between the two participants, a Student’s *t*-test was employed to calculate the *p*-values of each protein and the ones with *p* ≤ 0.05 were considered statistically significant. To further refine the dataset and account for variations, a Log2 fold-change calculation was applied to each protein. To accommodate the inherent uncertainty in protein abundance changes observed in the two participants, proteins with a fold-change (PR vs. PD) ≥ 2 or ≤ 0.5 were retained. Subsequently, the gene symbols of the filtered proteins, based on both *p*-values and fold-change criteria, were selected for Gene Ontology (GO) and Kyoto Encyclopedia of Genes and Genomes (KEGG) pathway-enrichment analysis.

### 4.6. Data Calculation and Visualization

The statistical function module named ‘stats’ in SciPy (https://scipy.org/) (accessed on 14 October 2023), which provides fundamental algorithms for scientific computing, was imported into Python for conducting hypothesis testing. Heatmaps were generated and clustered based on *p*-values using Excel. Box plots displayed the three quartile values of the distribution, including extreme values, and the corresponding significance markers were annotated according to *p*-values obtained from the Mann–Whitney U test. Strip plots were incorporated into box plots to present the original data for each individual. The GO and KEGG pathway enrichments were analyzed and the results were drawn into figures automatically by Metascape [39] (http://metascape.org) (accessed on 14 October 2023).

## 5. Conclusions

In our study, through a comprehensive array of statistical analyses conducted on pertinent datasets from the PPMI, we made several noteworthy observations regarding the impact of 90 PD-risk SNPs on urinary BMP levels. Specifically, among these SNPs, mutations in 9 of them were found to elevate BMP levels, 25 of them were associated with decreased BMP levels, 52 of them exhibited no significant changes in BMP levels within each cohort, and the remaining 4 of them had varying impacts across different cohorts and with different mutations. Our work demonstrated that variants rs34637584 ^G/A^ and rs34637584 ^A/A^ (LRRK2 G2019S) were the primary genetic factors elevating urinary BMP levels in the PD cohort compared to healthy controls; on the contrary, variants rs76763715 ^T/C^ and rs76763715 ^C/C^ (GBA1 N370S) mainly decreased the levels in the prodromal cohort compared to rs76763715 ^T/T^. These findings revealed the complexity between genetic variants and urinary BMP levels in different cohorts, challenging the notion of attributing higher urinary BMP levels to sole gene mutation.

A significant revelation from our study was the dramatic increase in BMP levels among patients carrying rs76763715 ^T/T^ within the prodromal cohort compared to the PD cohort. In another words, variant rs76763715 ^T/T^ increased the urinary BMP levels in the prodromal cohort. This unusual phenomenon ultimately caused higher BMP levels in the prodromal than in the PD cohort. Furthermore, it was crucial to acknowledge the considerable individual variations in BMP levels among patients. Even Box–Cox transformation failed to adhere the data to a normal distribution. Thus, the urinary BMP levels were not incremental alone with PD progression as common biomarkers were.

In brief, our findings cast doubt on the utility of elevated urinary BMP levels alone as reliable biomarkers for either diagnosing PD progression or identifying gene mutations. However, they held promise as supplementary indicators for facilitating early diagnosis and guiding disease management strategies. Additionally, the GO and KEGG pathway enrichment with proteomics data indicated the possible mechanism for unexpected elevated urinary BMP levels in prodromal patients, which might underlie the special material-transport pathways.

## 6. Innovations and Limitations of Our Work

Our study introduced several key innovations: 

(1) Comprehensive investigation of PD-risk SNPs: We conducted an extensive analysis involving all 90 PD-risk SNPs, assessing their impact on urinary BMP levels across 953 participants in distinct cohorts, including healthy controls, the prodromal cohort, and the PD cohort. This sample size significantly surpassed previous studies exploring the relationship between gene mutations and urinary BMP levels. Notably, we identified rs34637584 (LRRK2) and rs76763715 (GBA1) as the two most influential SNPs out of 90 SNPs, whose variants result in significant changes in urinary BMP levels.

(2) Exploration of non-mutated SNP variants: Our study uniquely considered the non-mutated variants of SNPs, particularly rs34637584 ^G/G^ (LRRK2) and rs76763715 ^T/T^ (GBA1), in both prodromal and PD patients. Surprisingly, we discovered that prodromal patients carrying rs76763715 ^T/T^ (GBA1) exhibited unexpectedly higher urinary BMP levels than those with rs76763715 ^T/C^ and rs76763715 ^C/C^ (GBA1 N370S) in this cohort. This revelation had not been previously reported in similar publications.

However, there were important limitations to consider:

(1) Data source limitations: All analyses relied on pre-existing data from the PPMI, which might contain inherent data gaps or a structure that did not fully align with our research objectives. For instance, the limited number of participants with rs34637584 ^A/A^ and rs76763715 ^C/C^ variants in the healthy control and prodromal groups (fewer than three) restricted our ability to draw definitive conclusions, leaving these aspects unaddressed. Moreover, due to data constraints, proteomics comparisons were limited to single samples from each cohort in the prodromal and PD groups, as only these individuals shared the same variants of rs34637584 (LRRK2) and rs76763715 (GBA1), which were established to be highly relevant to urinary BMP levels.

(2) Accuracy of random forest regression: While random forest regression was the most suitable method for our study, the complex interactions among numerous SNPs posed challenges in improving accuracy. Potential crosstalk among SNPs contributed to variations in results compared to individual SNP *p*-value computations.

(3) Limited scope of investigated genes: Our study focused exclusively on the 90 SNPs verified by Nalls et al. explaining 16–36% of the heritable risk of PD [28], while other common PD-risk genes, such as PRKN, PINK1, and VPS35, were not investigated. Expanding the scope to encompass these additional genes could provide a more comprehensive understanding of the genetic factors influencing BMP levels in PD.

(4) The definition of the prodromal cohort: In this study, the prodromal cohort defined by PPMI did not follow the research criteria of the Movement Disorder Society, which used an evidence-based methodology to statistically estimate the likelihood of prodromal PD. This might lead to bias in the categorization of participants.

## Figures and Tables

**Table 1:** The age and sex characteristics, and the overall urinary BMP features of participants.

Cohort	HC *n* = 190	PR *n* = 210	PD *n* = 554	*p*-Value ofHC vs. PR	*p*-Value ofHC vs. PD	*p*-Value ofPR vs. PD
Female number, %	67, 35.26%	123, 58.57%	215, 38.81%	−	−	−
Age (years),mean ± SD	60.83 ± 11.18	62.61 ± 7.30	62.40 ± 9.63	0.4590 ^a^	0.8893 ^a^	0.4590 ^a^
*p*-value of age between sex	0.0549 ^a^	0.4051 ^a^	0.9245 ^a^	−	−	−
*p*-value of total di-18:1-BMP between sex	0.8208 ^b^	0.8559 ^b^	0.1410 ^b^	−	−	−
*p*-value of total di-22:6-BMP between sex	0.4892 ^b^	0.4892 ^b^	0.0577 ^b^	−	−	−
*p*-value of 2,2’ di-22:6-BMP between sex	0.2588 ^b^	0.2398 ^b^	0.1739 ^b^	−	−	−
total di-18:1-BMP, mean ± SD	4.99 ± 4.23	13.53 ± 13.92	8.05 ± 9.96	<0.0001 ^b^	<0.0001 ^b^	<0.0001 ^b^
total di-22:6-BMP, mean ± SD	13.83 ± 13.02	56.94 ± 45.22	29.83 ± 38.79	<0.0001 ^b^	<0.0001 ^b^	<0.0001 ^b^
2,2’ di-22:6-BM, mean ± SD	9.11 ± 9.70	43.56 ± 37.24	22.07 ± 31.07	<0.0001 ^b^	<0.0001 ^b^	<0.0001 ^b^

In adherence to rigorous statistical practices, the *p*-values derived from pairwise comparisons were adjusted via Bonferroni correction due to multiple comparison. The critical threshold of significance was α = 0.0167 after Bonferroni correction. Note: HC—cohort of healthy controls; PR—cohort of prodromal stage; PD—cohort diagnosed with PD; SD—standard deviation; ^a^ Computed with Levene’s test and F-test in one-way ANOVA following Box–Cox transformation; ^b^ Computed with Mann–Whitney U test employing a ‘two-sided’ hypotheses approach after Box–Cox transformation.

**Table 2:** The partial correlation analysis to age, sex, cohort, rs34637584, rs76763715, and the urinary BMP levels by SPSS.

Covariates			Cohort	Total di-18:1-BMP	Total di-22:6-BMP	2,2’ di-22:6-BMP	rs34637584 (LRRK2)	rs76763715 (GBA1)
Sex and age	Cohort	corr	1.000	0.033	0.046	0.051	0.073	−0.023
		*p*	.	0.367	0.201	0.162	0.044	0.522
		df	0	758	758	758	758	758
	Total di-18:1-BMP	corr	0.033	1.000	0.502	0.509	0.412	−0.108
		*p*	0.367	.	0.000	0.000	0.000	0.003
		df	758	0	758	758	758	758
	Total di-22:6-BMP	corr	0.046	0.502	1.000	0.986	0.680	−0.143
		*p*	0.201	0.000	.	0.000	0.000	0.000
		df	758	758	0	758	758	758
	2,2’ di-22:6-BMP	corr	0.051	0.509	0.986	1.000	0.681	−0.148
		*p*	0.162	0.000	0.000	.	0.000	0.000
		df	758	758	758	0	758	758
	rs34637584(LRRK2)	corr	0.073	0.412	0.680	0.681	1.000	−0.220
		*p*	0.044	0.000	0.000	0.000	.	0.000
		df	758	758	758	758	0	758
	rs76763715(GBA1)	corr	−0.023	−0.108	−0.143	−0.148	−0.220	1.000
		*p*	0.522	0.003	0.000	0.000	0.000	.
		df	758	758	758	758	758	0

Note: corr—correlation; *p*—significance (two-tailed); df—degree of freedom.

## Data Availability

The original data used in this study is available on PPMI (https://www.ppmi-info.org/) (accessed on 12 August 2022). All figures, tables, and codes generated in this study are available from the corresponding author upon request.

## Supplementary Materials

The following supporting information can be downloaded at: https://www.mdpi.com/article/10.3390/ijms25042286/s1.
