# Supplementary material for: The Impact of 90 Parkinson’s Disease-Risk Single Nucleotide Polymorphisms on Urinary Bis(monoacylglycerol)phosphate Levels in the Prodromal and PD Cohorts
Source: Int J Mol Sci. 2024 Feb 14;25(4):2286. doi: 10.3390/ijms25042286 (PMC10889274; doi:10.3390/ijms25042286)
Supplement: Supplementary file 1 [file ijms-25-02286-s001.zip › Supplymental Figures.pdf]

Figure S1

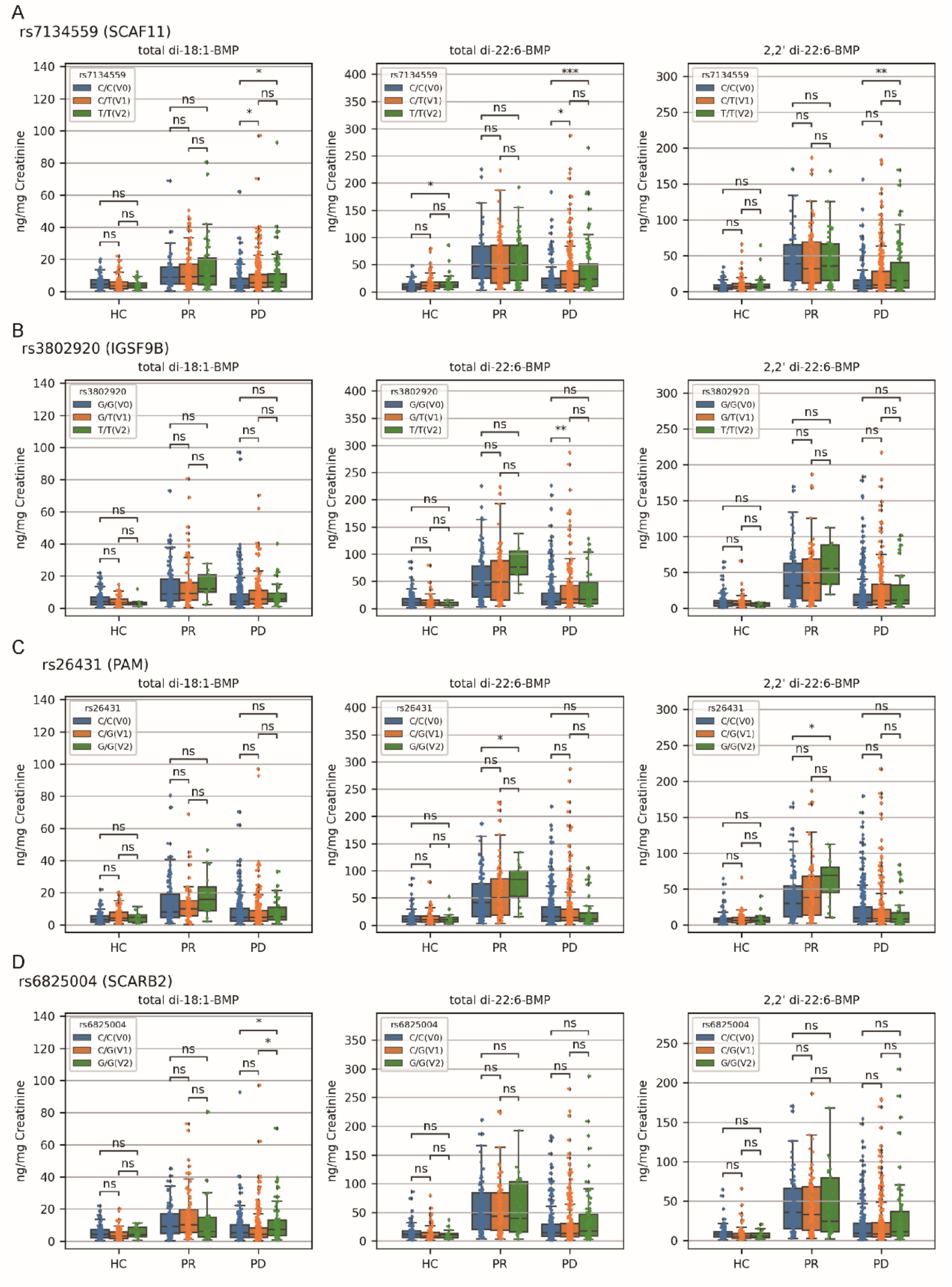

Figure S1. The four examples of SNPs whose variants increased the urinary BMP levels. Participants of each cohort were grouped by the variant they carrying, and their BMP levels after Box-Cox transformation were subjected to comparison using the M-W test with 'less' hypotheses when the number of participants in both groups were at least 3. All the computed p-values were adjusted with Bonferroni correction. HC: healthy controls, PR: the prodromal cohort, PD: the PD cohort; V0: the non-mutated homozygous variant, V1: the mutated heterozygous variant, V2: the mutated homozygous variant; ns: p>0.05, *: p≤0.05, **: 0.05<p≤ 0.001, ***: 0.0001≤p≤0.001, ****: p≤0.0001.

Figure S2

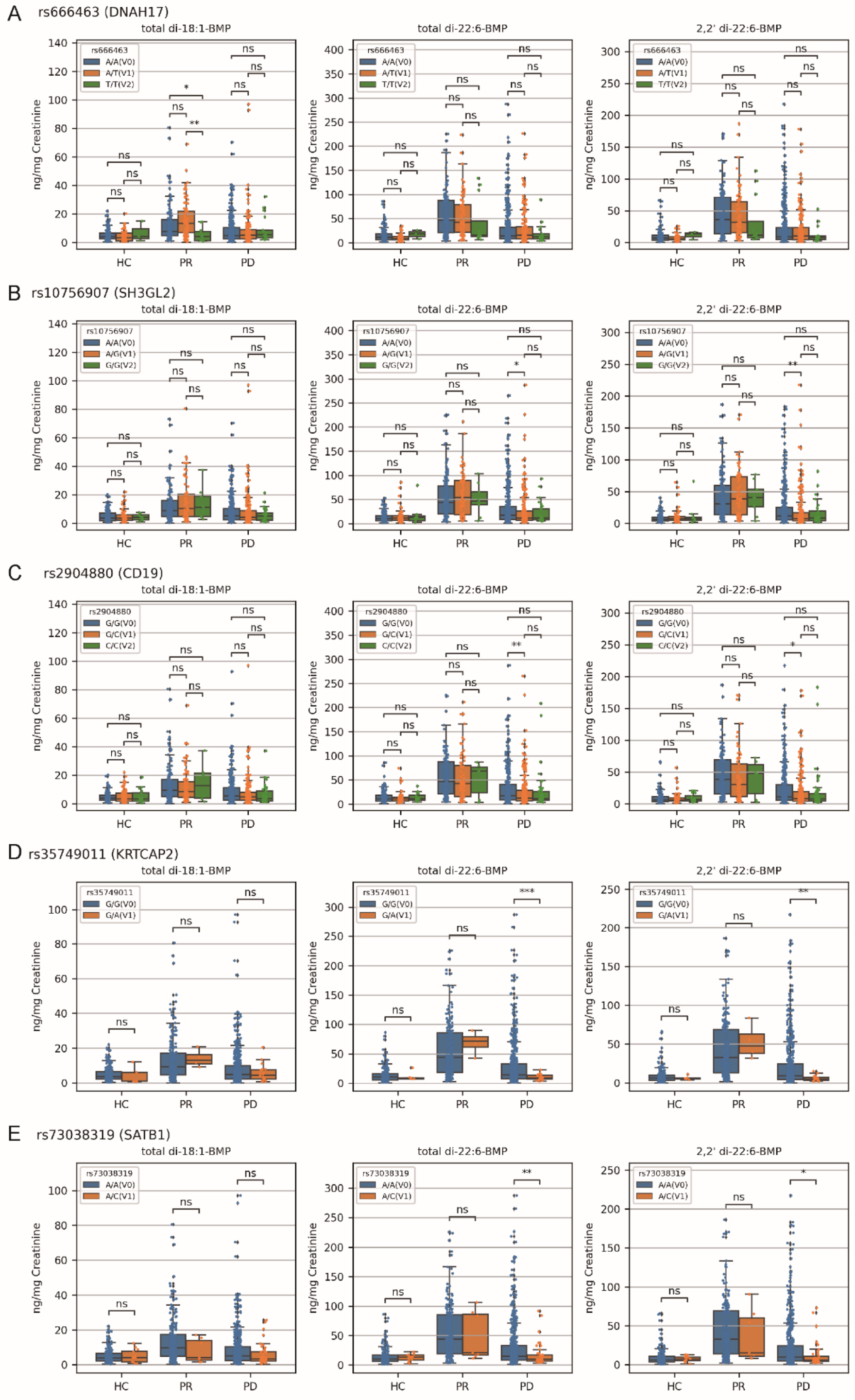

Figure S2. The five examples of SNPs whose variants decreased the urinary BMP levels. Participants of each cohort were grouped by the variant they carrying, and their BMP levels after Box-Cox transformation were subjected to comparison using the M-W test with 'greater' hypotheses when the number of participants in both groups were at least 3. All the computed p-values were adjusted with Bonferroni correction. HC: healthy controls, PR: the prodromal cohort, PD: the PD cohort; V0: the non-mutated homozygous variant, V1: the mutated heterozygous variant, V2: the mutated homozygous variant; ns: p>0.05, *: p≤0.05, **: 0.05<p≤ 0.001, ***: 0.0001≤p≤0.001, ****: p≤0.0001.

Figure S3

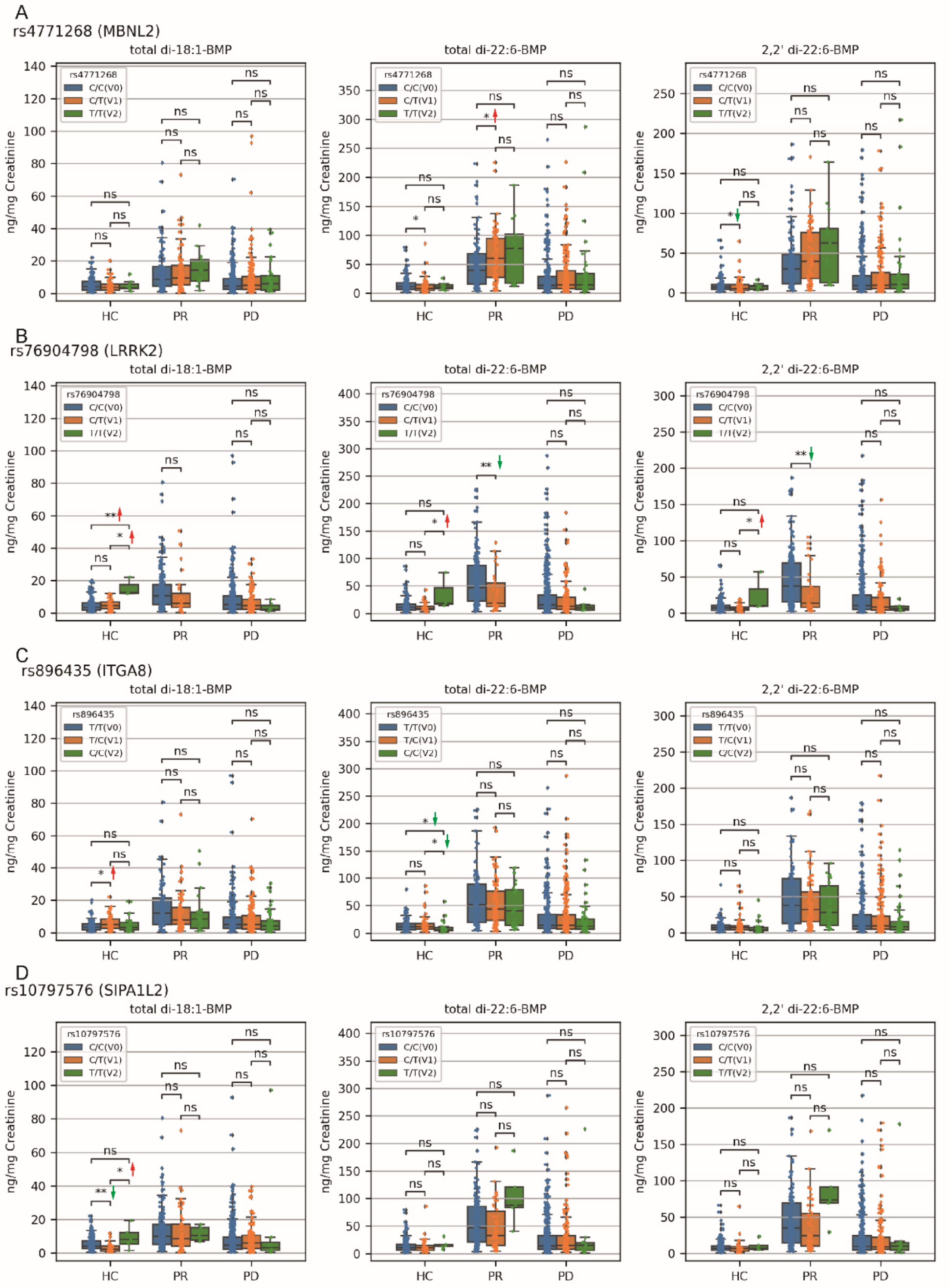

Figure S3. The five SNPs whose variants had varying impacts across different cohorts and with different mutations. Participants of each cohort were grouped by the variant they carrying, and their BMP levels after Box-Cox transformation were subjected to comparison using the M-W test successively with ‘less’ (the red arrows indicated significantly increase) and ‘greater’ (the green arrows indicated significantly decrease) hypotheses when the number of participants in both groups were at least 3. All the computed p-values were adjusted with Bonferroni correction. HC: healthy controls, PR: the prodromal cohort, PD: the PD cohort; V0: the non-mutated homozygous variant, V1: the mutated heterozygous variant, V2: the mutated homozygous variant; ns: p>0.05, *: p≤0.05, **: 0.05<p≤ 0.001, ***: 0.0001≤p≤0.001, ****: p≤0.0001.

Figure S4

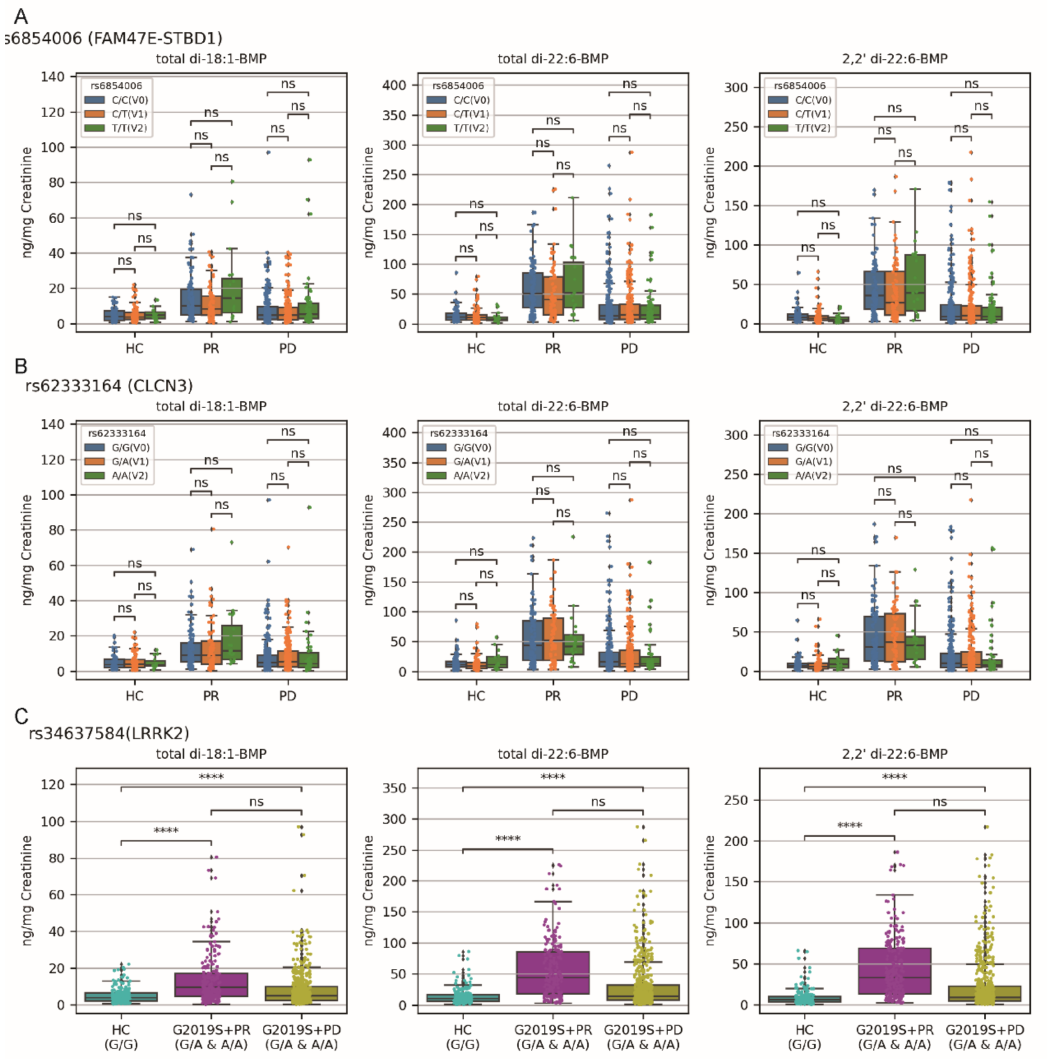

Figure S4. The SNPs related to urinary BMP levels change in PD cohort. (A) and (B) The second and third SNPs contributing to BMP levels in PD cohort identified by random forest regression actually showed no significant change across their variants. (C) LRRK2 G2019S (rs34637584 G/A and A/A) increased BMP levels in both prodromal and PD cohort compared to healthy controls. Boxplots were presented in a similar format to Merchant et al.'s work. All computed p-values were adjusted with Bonferroni correction. HC: healthy controls, PR: the prodromal cohort, PD: the PD cohort; V0: the non-mutated homozygous variant, V1: the mutated heterozygous variant, V2: the mutated homozygous variant; ns: $p>0.05$, *: $p\leq 0.05$, **: $0.05<p\leq 0.001$, ***: $0.0001\leq p\leq 0.001$, ****: $p\leq 0.0001$.

Figure S5

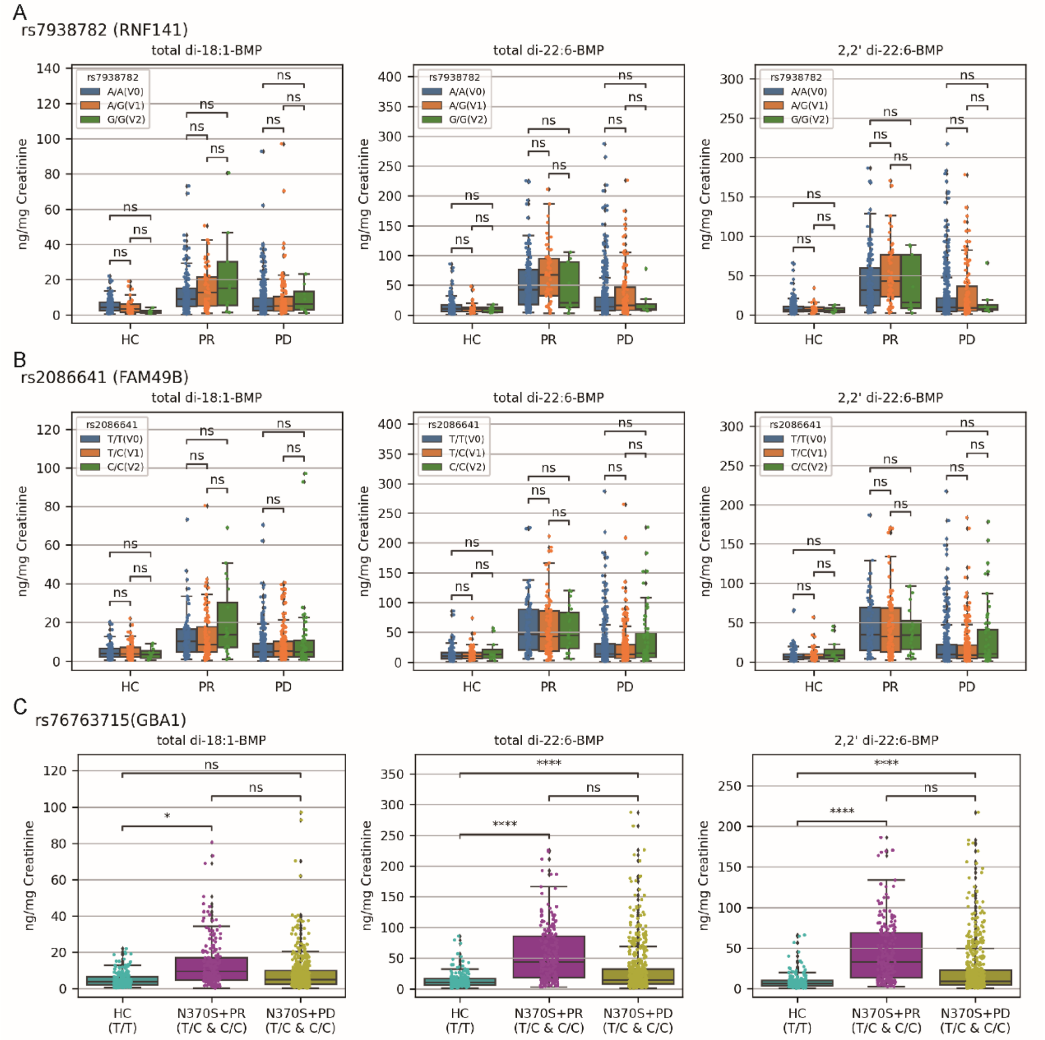

Figure S5. The SNPs related to urinary BMP levels change in prodromal cohort. (A) and (B) The second and third SNPs contributing to BMP levels in prodromal cohort identified by random forest regression actually showed no significant change across their variants. (C) GBA1 N370S (rs76763715 T/C and C/C) increased BMP levels in both prodromal and PD cohort compared to healthy controls. Boxplots were presented in a similar format to Merchant et al.'s work. All computed p-values were adjusted with Bonferroni correction. HC: healthy controls, PR: the prodromal cohort, PD: the PD cohort; V0: the non-mutated homozygous variant, V1: the mutated heterozygous variant, V2: the mutated homozygous variant; ns: p>0.05, *: p≤0.05, **: 0.05<p≤ 0.001, ***: 0.0001≤p≤0.001, ****: p≤0.0001.
